# Necrotic and inflammatory changes in metal-on-metal resurfacing hip arthroplasties: Relation to implant failure and pseudotumor formation

**DOI:** 10.3109/17453670903473016

**Published:** 2009-12-04

**Authors:** Gayana Mahendra, Hemant Pandit, Karolina Kliskey, David Murray, Harinderjit Singh Gill, Nick Athanasou

**Affiliations:** Nuffield Department of Orthopaedic Surgery, University of Oxford, Nuffield Orthopaedic Centre, Headington, Oxford, UK

## Abstract

**Background** Necrosis and inflammation in peri-implant soft tissues have been described in failed second-generation metal-on-metal (MoM) resurfacing hip arthroplasties and in the pseudotumors associated with these implants. The precise frequency and significance of these tissue changes is unknown.

**Method** We analyzed morphological and immunophenotypic changes in the periprosthetic soft tissues and femoral heads of 52 revised MoM arthroplasties (fracture in 21, pseudotumor in 13, component loosening in 9, and other causes in 9 cases).

**Results** Substantial necrosis was observed in the periprosthetic connective tissue in 28 of the cases, including all pseudotumors, and 5 cases of component loosening. A heavy, diffuse inflammatory cell infiltrate composed mainly of HLA-DR+/CD14+/CD68+ macrophages and CD3+ T cells was seen in 45 of the cases. Perivascular lymphoid aggregates composed of CD3+ cells and CD20+ B cells were noted in 27 of the cases, but they were not seen in all cases of component loosening or pseudotumors. Plasma cells were noted in 30 cases. Macrophage granulomas were noted in 6 cases of component loosening. In the bone marrow of the femoral head, a macrophage and T cell response was seen in 31 of the cases; lymphoid aggregates were noted in 19 of the cases and discrete granulomas in 1 case.

**Interpretation** Our findings indicate that there is a spectrum of necrotic and inflammatory changes in response to the deposition of cobalt-chrome (Co-Cr) wear particles in periprosthetic tissues. Areas of extensive coagulative necrosis and a macrophage and T lymphocyte response occur in implant failure and pseudotumors, in which there is also granuloma formation. The pathogenesis of these changes is uncertain but it may involve both a cytotoxic response and a delayed hypersensitivity (type IV) response to Co-Cr particles.

## Introduction

Second generation metal-on-metal (MoM) hip resurfacing is a recent development in hip arthroplasty. Although early clinical results of second generation MoM hip replacement were encouraging (Amstuz et al. 2004a, Treacy et al. 2005, Back et al. 2005, Pollard et al. 2006, Hing et al. 2007), a number of complications have been reported including thinning of the femoral neck, avascular necrosis, femoral neck fracture, implant loosening, metal ion release and hypersensitivity, and the formation of inflammatory pseudotumors (Capello et al. 1978, Amstuz et al. 2004b, Park et al. 2005, Shimmin et al. 2005, Boardman et al. 2006, Jacobs et al. 2006, Pandit et al. 2008).

The MoM bearing surface is made from high-carbon, cobalt-chromium-molybdenum alloy. This MoM combination produces less volumetric wear but results in the release of a much larger number of very small (nanometer-sized) particles compared to metal-polyethylene arthroplasties. Analysis of failed first-generation and second-generation MoM resurfacing arthroplasties has led to the identification of a number of histological changes arising from the deposition of these tiny metal particles in peri-implant tissues (Doorn et al. 1996, Davis et al. 2005, Willert et al. 2005, Korovessis et al. 2006). These include a pronounced macrophage response to wear particles, a perivascular lymphocytic infiltrate, and tissue necrosis. The necrotic and inflammatory changes seen in peri-implant tissues in response to cobalt-chromium (Co-Cr) metal wear debris are thought to be due to either cytotoxicity or to a delayed hypersensitivity reaction (Hallab et al. 2001, Willert et al. 2005, Keegan et al. 2007).

The significance of the necrosis and inflammation seen in periprosthetic tissues and bone around second-generation MoM arthroplasties has not been fully established. Necrosis and inflammation have been noted not only in failed MoM hip resurfacing arthroplasties (Doorn et al. 1996) but also in the recently described pseudotumors that develop around some MoM implants (Boardman et al. 2006, Pandit et al. 2008). In this study, we have examined the extent of necrosis and inflammation in peri-implant soft tissues and bone for a large series of revised second-generation MoM hip implants in order to determine the frequency, nature, and significance of these changes.

## Patients and methods

Our study included 50 patients (29 females) with a mean age of 55 (25–75) years at the time of revision surgery, which was carried out between 2001 and 2007 (Table). The primary operation was hip resurfacing in all patients. In 2 patients, the revision surgery was performed on both hips. The commonest cause of revision surgery was fracture (21). Other causes included periarticular pseudotumor formation (13), component loosening (9), instability or recurrent dislocation (3), unexplained pain (5), and avascular necrosis (1). The mean time to revision surgery was 20 months (range: 1 week to 75 months). All cases initially underwent arthroplasty for osteoarthritis; none of the cases had microbiological or histological evidence of acute infection and cases of rheumatoid disease were excluded. In 30 cases, samples of periprosthetic soft tissue (including the pseudocapsule and the acetabular and femoral pseudomembrane) and the revised femoral head were examined. In 14 cases only the femoral head was examined and in 8 cases only the periprosthetic tissue was examined.

**Table:** Details of revised hip resurfacings including patient demographics and duration “in situ” for each group of patients

	No. of hips	Primary implant	Mean age [SD] (range)	Sex	Mean time to revision in months [SD] (range)
Pseudotumor	13	8 BHR, 4 Conserve Plus, 1 Cormet	55 [13] (30–69)	13 F	34 [23] (9–79)
Fracture	21	13 BHR, 8 Conserve Plus	57 [11] (29–76)	8 F, 13 M	7 [11] (0.6–42)
Component loosening	9	4 BHR, 3 Conserve Plus, 2 Cormet	59 [13] (30–76)	6 F, 3 M	27 [15] (1.4–45)
Other	9	5 BHR, 4 Conserve Plus	60 [8] (46–66)	4 F, 3 M	30 [29] (0.1–76)

### Histological analysis of periprosthetic soft tissues and bone

5-µm-thick tissue sections of the pseudocapsule and pseudomembrane were analyzed for extent and type of tissue necrosis, the nature and degree of inflammation, and the presence of wear debris. The degree of inflammation, including lymphocyte and macrophage infiltration, was assessed using the semiquantitative grading system of Willert and Semlisch (1997) in which none, few, many, abundant, and excessive inflammatory cell infiltration is scored as grade 0, 1, 2, 3, or 4. The presence of lymphoid aggregates was noted with regard to their depth of location (i.e. superficial or deep) and whether they were perivascular in distribution. The presence of plasma cells, granulomas, eosinophils, and neutrophil polymorphs within the inflammatory infiltrate was also noted. The microanatomical surface layer of the periprosthetic tissue was assessed to determine whether there was an intact cellular lining or whether there was disruption of the lining and the surface was covered with fibrin (Keegan et al. 2007). The nature and degree of inflammation in cancellous bone was similarly assessed. Cancellous bone was also examined for the presence or absence of osteonecrosis and the presence of wear debris.

### Immunohistochemical analysis

The inflammatory cell infiltrate in periprosthetic tissues and bone from 18 representative cases was analyzed by immunohistochemistry using monoclonal antibodies F7.2.38, NCL-L-CD4-368, C8/144B, L26, CD14-223, KPI, 120507, M7077, and CR3/43 directed against T lymphocytes (CD3, CD4, CD8), B lymphocytes (CD20), macrophages (CD14, CD68), plasma cells, dendritic cell antigens, and HLA-DR, respectively.

## Results

### Histological analysis of periprosthetic soft tissues following resurfacing arthroplasty

Similar histological features were noted in the pseudocapsule and the pseudomembrane of all revised MoM arthroplasties. PMMA wear debris was commonly seen in periprosthetic tissues. Individual metal Co-Cr particles, being submicron in size, could not be visualized by light microscopy, but scattered fine, black particles—presumed to be aggregates of Co-Cr particles—were seen within macrophages and in extracellular connective tissue. The surface of the pseudocapsule or pseudomembrane was intact and covered by a synovial lining in only 4% of cases; in the remaining cases the surface was ulcerated, and in 15 of these cases the surface was covered by fibrinous material.

**Figure 1.**
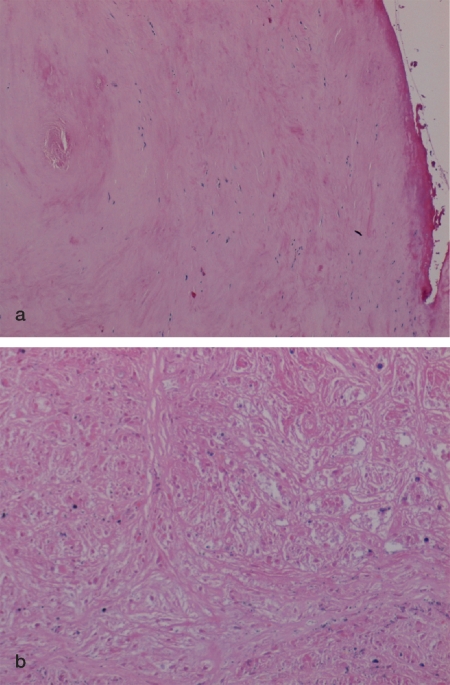
Necrosis in peri-implant soft tissues of an MoM hip resurfacing. (a) Necrosis of superficial fibrous tissue that is covered by fibrin in a case of component loosening. (b) Coagulative necrosis in which ghost-like outlines of plump macrophages are evident in a case of pseudotumor.

Connective tissue necrosis was observed in the superficial or deep zones of periprosthetic soft tissues in all cases; this necrotic change varied considerably in amount, although it was more prominent in cases where the implant had been in situ for more than 12 months. Necrosis was a significant feature (more than 25% tissue necrosis in a single histological section of pseudocapsule or pseudomembrane) in 28 of the cases examined, including all cases of pseudotumour (Figure 1a). Significant necrosis was seen in 30 cases of component loosening, but necrosis was less marked in cases of fracture, with the exception of two cases where the implants had been in situ for 15 months and 36 months. In all cases of pseudotumor formation and focally in 2 cases of component loosening, ghost-like outlines of macrophages that in many cases appeared to lie in small (granuloma-like) aggregates were seen (Figure 1c). Focal areas of cystic degeneration were also noted within areas of extensive necrosis in pseudotumor cases.

**Figure 2.**
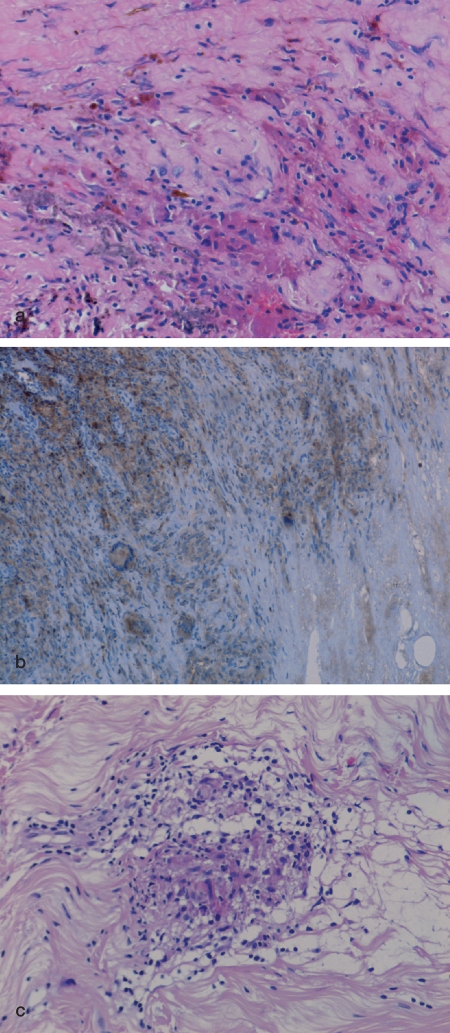
Inflammation in the pseudomembrane from a case of component loosening showing (a) macrophage infiltration and metal particles (arrowed); (b) macrophage and giant cell expression of CD68; and (c) discrete granuloma formation.

**Figure 3.**
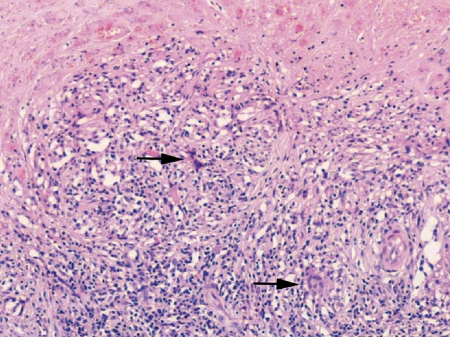
Macrophage and giant cell (arrowed) granulomatous response around an area of coagulative necrosis (top) in a case of MoM-implant associated pseudotumor.

An inflammatory cell infiltrate composed mainly of macrophages and lymphocytes was noted in the pseudocapsule and pseudomembrane of 86% revised MoM implants. Macrophages formed a significant component of the inflammatory response to wear particles in periprosthetic soft tissues. This macrophage infiltrate (of grades 1–3) was composed of plump mononuclear cells and macrophage polykaryons that expressed CD 68, HLA-DR, and CD14 (Figure 2). Discrete granulomas, composed of HLA-DR+/CD68+ macrophages and giant cells, were seen in cases of pseudotumor and in three cases of component loosening. In cases of pseudotumor, granulomatous response was seen around areas of necrosis (Figure 3).

**Figure 4.**
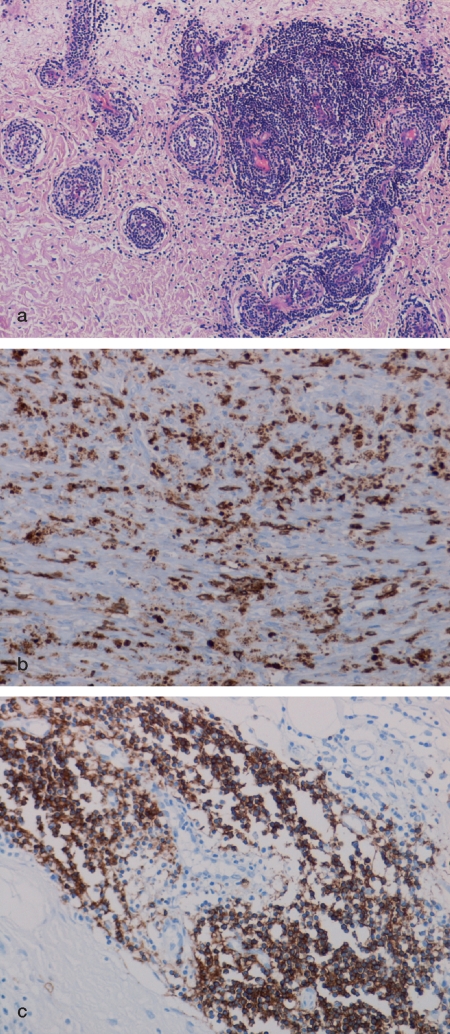
Pseudomembrane from a case of MoM implant component loosening showing (a) numerous perivascular lymphoid aggregates and a diffuse lymphocyte infiltrate; (b) expression of CD3 by lymphocytes in the diffuse inflammatory cell infiltrate; and (c) CD20-expressing B lymphocytes in lymphoid aggregates.

A diffuse lymphocytic infiltrate in superficial and deep fibrous tissue (grade 1–3) was commonly seen in periprosthetic soft tissues in revised MoM cases (Figure 4). Scattered perivascular lymphoid aggregates were also seen in 27 cases, mainly in deep periprosthetic tissue. In 19 cases, 3 or more lymphoid aggregates were noted per high-power field (HPF). Lymphoid aggregates were seen in 41 cases of component loosening and in 30 of the pseudotumor cases. Immunohistochemistry showed that the diffuse lymphocytic infiltrate was composed mainly of CD3+ T cells with few or no CD20+ B lymphocytes. CD4+ and CD8+ cells were noted in equal numbers. The lymphoid aggregates contained CD3+ T lymphocytes and CD20+ B lymphocytes. Plasma cells often scattered around lymphoid aggregates were noted in 30 cases; more than 10 plasma cells per HPF were seen in 14 cases. More than 2 eosinophil polymorphs per HPF were noted in 3 cases.

### Histological analysis of the femoral head bone following resurfacing hip arthroplasty

**Figure 5.**
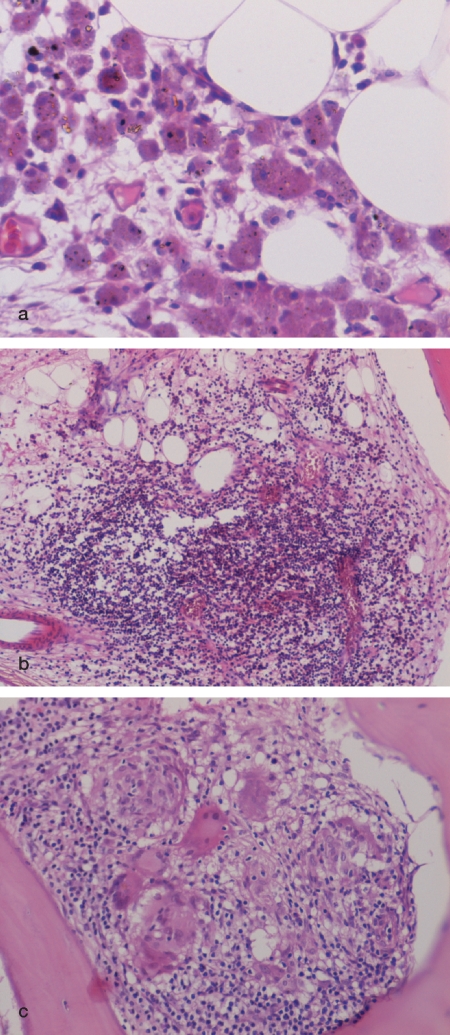
Femoral bone marrow from a revised MoM hip resurfacing showing (a) a collection of macrophages containing metal particles (arrowed); (b) a small lymphoid aggregate; and (c) a discrete macrophage and giant cell granuloma.

In 31 cases where revised femoral heads were available for analysis, there was a chronic inflammatory cell infiltrate in the bone marrow. This inflammatory infiltrate was composed mainly of CD68+/CD14+/HLA-DR+ macrophages, some of which contained metal particles, and lymphocytes (Figure 5a and b). A heavy (grade 2–3) lymphocytic infiltrate composed mainly of CD3+ T cells was noted in 8 cases; almost all of these cases had a grade 2–3 lymphocytic infiltrate in periprosthetic soft tissues as well. CD20+ B cells were seen in 19 cases. A macrophage and giant cell granuloma was noted in one case of prosthesis fracture (Figure 5c). Osteonecrosis, marrow fibrosis, remodeling change in bone trabeculae, and degenerative change in the marrow were noted in almost all femoral heads.

## Discussion

Our findings indicate that a spectrum of necrotic and inflammatory changes can be seen in peri-implant tissues of MoM arthroplasties and that these changes are most pronounced in cases of component loosening and pseudotumor formation. The pathogenesis of the necrosis and inflammation that occurs as a result of the deposition of Co-Cr wear particles in MoM peri-implant tissues is not certain, but our results suggest that both cytotoxicity and hypersensitivity to metal particles may play a role in inducing these changes.

Coagulative necrosis was seen in periprosthetic soft tissues of all failed MoM arthroplasties in our study. Vascular occlusion, either due to a vasculitis or a toxic effect on blood vessel walls associated with Co-Cr metal wear particle deposition (Evans et al. 1974, Winter 1974, Jones et al. 1975, Doorn et al. 1996, Willert and Semlisch. 1997), has been suggested as a cause of this tissue necrosis in long-term and short-term first- and second-generation MoM hip replacements. However, Brown et al. (1977) found no correlation between vascular occlusion and periprosthetic tissue necrosis, and experimental studies have found that synovial tissue necrosis following injection of Co-Cr particles into rat knee joints does not appear to correlate with vascular occlusion or inflammation (Howie and Vernon-Roberts 1988). In our series of cases, we found no morphological evidence to suggest an acute or chronic vasculitis, such as fibrinoid necrosis or destruction of the elastic lamina of small vessels. In addition, a perivascular lymphocytic infiltrate was not seen in all cases where there was tissue necrosis. Thus, our observations do not point to the necrosis in periprosthetic soft tissues being solely due to a vasculitis-type process.

The volumetric wear rate of MoM bearings is lower than that of metal-on-polyethylene bearings, but the number of particles generated from MoM bearings is much higher because the metal particles are considerably smaller. These Co-Cr particles have a high specific surface area, promoting the dissolution of these metal ions into surrounding tissues. These ions are known to induce apoptosis and necrosis of macrophages, the latter being seen particularly at high ion concentrations (Huk et al. 2004). Our finding of necrosis in revised MoM periprosthetic tissues, including those cases where relatively little associated inflammation was evident, would indicate that cytotoxicity cannot be discounted as a possible cause of peri-implant tissue necrosis. Ultrastructural studies have shown that metal particles phagocytosed by macrophages are transported to lysosomes; high concentrations of metal ions would be released in these structures, resulting in apoptosis and cell death with subsequent release of the phagocytosed metal (Salvati et al. 1993). The morphology of the necrotic and viable macrophage granulomas seen in pseudotumors, and in some cases of MoM component loosening, would be in keeping with this cytotoxic effect causing a vicious circle in which metal wear particle generation leads to macrophage recruitment, particle phagocytosis, apoptosis, and cell death with resultant release of metal particles—leading to further macrophage recruitment, and repetition of this process. Surface ulceration of the pseudocapsule and pseudomembrane around MoM implants may result from a similar process as the cells that line the pseudocapsule are mainly macrophage in phenotype; exposure of articular synovial tissue to Co-Cr debris is sufficient to provoke surface ulceration and a lymphocytic infiltrate in the absence of a loose prosthesis (Howie et al. 1988).

A perivascular lymphocyte and plasma cell reaction, “aseptic lymphocyte-dominated vascular associated lesion” (ALVAL), has been noted in periprosthetic soft tissues of MoM arthroplasties that have undergone early failure (Davis et al. 2005, Willert et al. 2005). It is considered to develop as a result of a delayed hypersensitivity response to Co-Cr metal wear debris. A diffuse macrophage and lymphocyte infiltrate was commonly seen in peri-implant MoM soft tissues and was grade 2–3 in degree in cases of pseudotumor and component loosening. An ALVAL response, however, was not seen in all revised MoM cases, including those defined as cases of implant failure due to component loosening. We did not observe the macrophage inclusions reported by Willert et al. (2005), but noted that macrophages and macrophage polykaryons formed discrete granulomas in some cases. Lymphocytes in failed surface arthroplasties were mainly CD3+ T lymphocytes, comprising a mixture of CD4+ and CD8+ cells. B lymphocytes and plasma cells were found predominantly in lymphoid aggregates. There was also a variable eosinophil polymorph and dendritic cell infiltrate, and some cases of component loosening contained discrete (sarcoid-like) granulomas as well as the typical necrotic granulomas found in pseudotumors. The presence of numerous lymphocytes, macrophages, eosinophil polymorphs, and antigen presenting cells, as well as granuloma formation, would be in keeping with a hypersensitivity-induced inflammatory response, mainly type IV in nature, occurring in periprosthetic soft tissues. The presence of a similar inflammatory cell infiltrate in the bone marrow of femoral heads retrieved from revised MoM arthroplasties indicates that a similar response to wear particles occurs in bone. Osteonecrosis of the femoral head has also been seen in many of these cases, a number of which presented with fracture (Little et al. 2005).

Our finding of peri-implant tissue necrosis and a macrophage response in all revised MoM cases, including those with little associated lymphocytic infiltrate, would be in keeping with the concept of phagocytosis of cytotoxic Co-Cr particles inducing the macrophage apoptosis and cell death seen in the necrotic areas of pseudotumors. It is well recognised that Co-Cr particles can induce activation and transformation of lymphocytes (Salvati et al. 1993), and a T lymphocyte response was seen in almost all revised MoM cases including cases of pseudotumor formation, where it was associated with a heavy macrophage infiltrate and granuloma formation. These morphological and immunological changes would be in keeping with a delayed (type IV) hypersensitivity response to metal particles playing a role in pseudotumor formation. A lymphocytic and necrotic granulomatous response can develop in cases of contact skin hypersensitivity in response to metal components of cheap jewellery, which often contain nickel or chromium; crossover hypersensitivity between these metals is known to occur (Casper et al. 2004). It has been suggested that the hypersensitivity response to metal components in this jewellery may explain the high frequency of pseudotumors in females. The hypersensitivity response to metals is probably variable but, when pronounced, it may be a major contributory factor to the necrosis and inflammation associated with component loosening and pseudotumor formation.

MG: data analysis and manuscript preparation; PH: provided clinical expertise, co-ordinated the project, and wrote the clinical aspects of the paper; KK: created the database, data entry and analysis; MD: conception and study design, highlighted the clinical problem, revision of manuscript; GHS:methodological expertise, revision of manuscript; AN: conception and study design, data collection and analysis, manuscript preparation.
